# Rethinking global statin guidelines for older adults in resource-diverse settings

**DOI:** 10.7189/jogh.15.03047

**Published:** 2025-12-05

**Authors:** Karima Chaabna, Ravinder Mamtani, Sohaila Cheema

**Affiliations:** Weill Cornell Medicine-Qatar, Institute for Population Health, Doha, Qatar

## Abstract

Statin guidelines for older adults are predominantly informed by evidence from high-income countries (HICs), making them less relevant in low- and middle-income countries (LMICs) with varying healthcare capacities. Identical patients may receive different recommendations depending on the geographic context, as seen in European Systematic Coronary Risk Evaluation 2 (SCORE2) and USA’s Predicting Risk of Cardiovascular Disease EVENTs (PREVENT) guidelines. LMICs often rely on the World Health Organization cardiovascular risk charts and implementation frameworks, such as the package of essential non-communicable disease interventions and HEARTS. While these frameworks are generally more feasible in resource-limited settings, they lack the clinical specificity of HIC-based guidelines. Emerging biological evidence challenges uniform cholesterol-lowering therapies in older adults. Polypharmacy, potential adverse effects, and the limited capacity for ongoing monitoring in many settings further complicate the net benefit of statin therapy in this population. These challenges underscore the need for context-sensitive, age-appropriate guidelines. We outline a context-sensitive approach to statin use in older adults and propose guiding principles to support more equitable, feasible, and clinically appropriate decision making. These include aligning treatment with functional status and prognosis, using fixed-dose combinations, and integrating statin use into broader primary care strategies through task-sharing and simplified protocols. To ensure meaningful cardiovascular disease prevention in ageing populations, global guidelines must evolve to reflect regional capacity, biological variation, and implementation.

Hypercholesterolemia is a major risk factor for atherosclerotic cardiovascular disease (CVD), a leading cause of death among older adults globally [1]. Clinical guidelines for statin use are based largely on evidence from high-income countries (HICs), which rely on risk prediction models included in the Systematic Coronary Risk Evaluation 2 (SCORE 2) from Europe [2] and Predicting Risk of Cardiovascular Disease EVENTs (PREVENT) [3] from the USA. Statin use is important in this context because evidence demonstrates that it significantly reduces the risk of major cardiovascular events, such as heart attack and stroke, by lowering low-density lipoprotein (LDL) cholesterol levels, which is the primary driver of the atherosclerotic process. Although both originate from HICs, these guidelines provide conflicting recommendations for identical patients across regions [2]. These regional discrepancies underscore the limitations of a one-size-fits-all approach, which fails to account for biological diversity and healthcare resource limitations, especially in low- and middle-income countries (LMICs). These challenges are critical for adults aged ≥60 years, who remain underrepresented in clinical trials informing the current guidelines [4]. This evidence gap is compounded in LMICs, where older adults are rarely included and where trial findings are often not generalisable due to differing health system capacities. Here, we examine how the statin guidelines may be disconnected from the realities of ageing populations and propose core principles for developing context-sensitive, feasible, and equitable recommendations.

## GLOBAL DISPARITIES IN STATIN GUIDELINES

Recent advances in CVD risk prediction have highlighted major inconsistencies in statin eligibility, whereby the same patient may qualify under USA guidelines (34%), but not under European guidelines (4%) [2,3], reflecting divergent thresholds and treatment philosophies. While USA models favour pharmacological prevention and European guidelines prioritise lifestyle changes and higher-risk cut-offs, many LMICs struggle to apply either approach due to limitations in data, infrastructure, and access [5]. These regional differences raise critical concerns about the equity and appropriateness of applying uniform statin guidance across diverse populations, health systems, and ageing trajectories.

These inconsistencies are more pronounced in adults aged ≥60 years – a group historically underrepresented in clinical trials [4]. While meta-analyses have suggested the potential benefits of cholesterol-lowering treatments in patients aged ≥75 years [6], their applicability across diverse populations and healthcare systems remains unclear. USA guidelines recommend considering statin discontinuation in patients aged ≥75 years [7], emphasising individualised care and regular monitoring, which are often not feasible in global settings. Statin use in older adults should account for health status, competing risks, functional status, frailty, polypharmacy, and life expectancy. For adults aged 60–74 years, the focus should be on targeting prevention. For those aged ≥75 years, decisions should be guided by prognosis, including whether to continue therapy or deprescribing.

## BIOLOGICAL DIVERSITY AND CHOLESTEROL TARGETS

The current guidelines assume that lowering LDL cholesterol reduces CVD risk. However, this correlation is more complex in adults aged ≥60 years, with some studies finding lower mortality among those with higher LDL levels [8]. High cholesterol levels are also common in centenarians [8,9], with no evidence of increased CVD incidence. In contrast, normal and even beneficial cholesterol levels may be misclassified as high-risk in some populations [9], particularly in regions where genetic, dietary, and environmental factors can influence lipid metabolism and cardiovascular risk differently. Also, cholesterol levels vary among populations. The INTERHEART study showed that populations like South Asian (150 mg/dL), Latin American (155 mg/dL), and Arab/Persian individuals (153 mg/dL) had similar or lower non-HDL cholesterol levels (recognised as an independent risk factor for CVD) than Europeans (158 mg/dL), yet experienced higher CVD risk [10]. Moreover, current guidelines [7,11] are largely based on younger populations from high-income countries, increasing the risk of overdiagnosis and overtreatment of older adults worldwide [8]. These findings underscore the need for treatment approaches that account for biological and regional variations.

The management of hyperlipidaemia in older adults must consider the side effects of statin therapy, such as muscle pain and increased diabetes risk [12,13]. Additionally, given the multiple comorbidities and polypharmacy, the risk-benefit assessment of statin therapy is complicated in older patients. Therefore, differentiating between primary and secondary prevention strategies is essential for effective statin use. While secondary prevention is strongly supported by evidence and is highly cost-effective, primary prevention in older adults requires more nuanced, individualised approaches to avoid overtreatment and ensure optimal allocation of limited healthcare resources.

## IMPLEMENTATION CHALLENGES IN DIVERSE HEALTHCARE SETTINGS

Ongoing trials such as the Statins in Reducing Events in the Elderly trial (STAREE; NCT02099123) and the Pragmatic Evaluation of Events and Benefits of Lipid-Lowering in the Elderly trial (PREVENTABLE; NCT04262206), conducted in high-resource settings, promise more evidence on statin use in older populations but have limited applicability in LMICs. A cross-sectional analysis of 45 LMICs found that only 24.2% of men and 41.6% of women with high CVD risk received guideline-recommended medications [14], highlighting the gap between clinical recommendations and healthcare practices.

Health system constraints, such as limited access to lipid testing, irregular statin supply, overburdened clinicians, insufficient provider training, lack of guideline familiarity, and absence of decision-support tools, impede effective treatment in LMICs. Task sharing with trained nonphysician providers, supported by simplified protocols, offers a scalable strategy to improve access and adherence [15]. Real-world programmes, including India’s national programme for non-communicable diseases and South Africa’s nurse-led chronic disease management initiatives, show that statin delivery can be effectively integrated into primary care through community-based models. The World Health Organization (WHO) promotes an integrated approach through frameworks such as the HEARTS technical package and the package of essential non-communicable (PEN) disease interventions for primary healthcare. These initiatives combine lifestyle counselling, hypertension management, and essential medicines, including statins, under standardised, scalable protocols [16].

In low-resource settings, non-laboratory-based risk models, such as WHO risk charts, provide pragmatic alternatives to laboratory-dependent tools [17]. Incorporated into broader strategies, such as WHO PEN and HEARTS technical packages, these models rely on clinical indicators such as age, blood pressure, and smoking status. Yet they lack the granularity of tools like PREVENT or SCORE2, offering limited guidance for older adults or patients with complex conditions.

Although the WHO models reduce reliance on cholesterol testing at baseline, monitoring after statin initiation remains important for dose adjustment and safety. Point-of-care cholesterol testing may improve access [18], but requires reliable supply chains, adequate training, and regulatory oversight. Moreover, strengthening the diagnostic infrastructure remains a critical priority.

While generic statins have reduced drug costs, healthcare systems still face substantial indirect expenses associated with follow-up, healthcare provider time, and management of side effects. The inclusion of national essential medicine lists supports procurement and reimbursement [19], but does not ensure access. In many low-resource settings, supply chain gaps, financing issues, and stock-outs continue to limit their availability.

Fixed-dose combinations, like polypills containing a statin, offer a pragmatic solution. The Secondary Prevention of Cardiovascular Disease in the Elderly trial (SECURE; NCT02596126) and the Polypill for the prevention of cardiovascular disease trial (PolyIran; NCT01271985) have demonstrated reduced cardiovascular events among patients in various resource settings, supporting polypills as a scalable option. While modest *per capita* investments could avert millions of deaths [20], allocating resources remains a challenge in LMICs. Hence, guidelines must balance clinical evidence with economic feasibility to support sustainable CVD prevention in the ageing population.

## TOWARDS EQUITABLE CARDIOVASCULAR GUIDELINES

Statin guidelines must evolve to reflect biological diversity, local health system capacity, and the complexities of ageing. Hence, in collaboration with national governments and non-governmental organisations, the WHO should prioritise the development of updated, tiered recommendations that consider the above factors. Notably, the last WHO Guidelines for the Prevention of Cardiovascular Disease were published in 2007 and require updating to reflect the current evidence and emerging global health priorities.

Strengthening equitable statin use requires investments in region-specific implementation research, decision support tools, and workforce capacity [20]. A tiered approach could emphasise lifestyle interventions and generics in LMICs, while enabling advanced lipid therapies and precision medicine for well-resourced healthcare systems. Frailty, polypharmacy, and limited life expectancy must inform both initiation and deprescribing decisions in older adults, especially those aged ≥75 years. Incorporating geriatric assessment tools and patient preferences into guideline development is essential.

## POLICY IMPLICATIONS AND RECOMMENDATIONS

To address the limitations of current statin use in older adults, it is essential to adopt a context-sensitive framework that considers biological diversity and the healthcare system capacity (**Table 1**). First, the evidence base should extend beyond high-income countries, as studies have suggested that normal cholesterol levels differ across populations [8–10]. Second, guidelines must acknowledge resource constraints by adopting a tiered approach based on healthcare system capacity. In LMICs, this could involve using existing population-based data such as WHO risk charts, national STEPwise approach to non-communicable diseases risk factor surveillance (STEPS), and cohort studies, to define regionally appropriate risk thresholds, prioritise non-laboratory-based assessments, and guide cost-effective statin initiation strategies. This should include flexible recommendations and alternative strategies for resource-limited settings, such as different thresholds for initiating statin therapy and nonpharmaceutical interventions to reduce CVD risk. Statin use should move beyond a binary prescription approach toward a nuanced strategy that considers the local healthcare context, population characteristics, and competing health priorities. Greater autonomy within regional healthcare systems is crucial to adapt guidelines to specific needs.

**Table 1:** Comparison of statin guideline approaches for older adults: practical contrasts between conventional models and a context-sensitive framework

Dimension	HIC-centric approach, reference	Proposed context-specific framework. reference	In practical terms, reference
Evidence base	Primarily derived from high-income countries [4]	Inclusive of diverse populations, including LMICs contexts [8,9,20]	Conduct region-specific clinical trials, including older adults and leveraging local epidemiological data to inform guidelines better.
Cholesterol targets	Uniform targets mainly based on younger Western populations [7,10]	Population-specific, accounting for genetic and environmental variations [8,9]	Set cholesterol targets based on local population health data, considering genetic predispositions and dietary habits.
Risk assessment models	Laboratory-based models embedded in the clinical guidelines SCORE [2] and PREVENT [3]	Adaptive models that consider regional healthcare capabilities [14]; uses tiered or non-laboratory-based tools (*e.g.* WHO chart, HEARTS)	Maintain SCORE [2]/PREVENT [3] in HICs. Apply WHO risk charts in LMICs where laboratory testing is limited. Develop or adapt tools that incorporate region-specific risk factors and healthcare system capacity
Assessment of older adults	Often limited to age thresholds; comorbidities, frailty, and polypharmacy are under-addressed in clinical decision-making	Incorporates functional status, life expectancy, frailty, and competing risks into clinical decision-making	Use geriatric tools (*e.g.* clinical frailty scale, STOPP/START, prognosis calculators) to tailor statin decisions in adults aged ≥75 y.
Implementation infrastructure	Assumes regular laboratory testing, monitoring, and specialist oversight	Uses a tiered and flexible approach based on available infrastructure and monitoring capabilities [14,20]	Use frequent laboratory tests and specialist care in high-resource settings. Use point-of-care testing and risk tiering in LMICs.
Health workforce	Specialist-driven, limited use of task-sharing	Healthcare workforce deployed based on healthcare system capacity	Use frequent laboratory tests and specialist reviews for monitoring in high-resource settings. Deploy community health workers or mobile health solutions in LMICs to track adherence and manage side effects (task-sharing with trained health workers and decision tools)
Delivery strategies	Relies on physician-led, clinic-based care	Uses task-sharing, decision-support tools, and fixed-dose combinations	Expand nurse/pharmacist roles in LMICs. Deploy polypills for secondary prevention.
Economic considerations	Uniform medication recommendations	Resource-sensitive interventions tailored to local resources [14,20]	Prioritise the use of cost-effective generics and essential medicines while ensuring affordability through government subsidies or insurance schemes.
Side effect management	Generic approach	Population-specific risk assessment [12,13,21]	Assess factors such as polypharmacy, dietary interactions, and genetic predispositions when prescribing and monitoring statins.

HIC – high-income countries, LMICs – low- and middle-income countries, PREVENT – Predicting Risk of Cardiovascular Disease EVENTs, SCORE – Systematic Coronary Risk Evaluation, WHO – World Health Organization

Global health stakeholders, particularly the WHO and national health authorities [8–10] should employ a flexible framework. This approach should prioritise low-cost lifestyle interventions and generic medications in resource-limited settings while enabling advanced lipid management in well-resourced health systems [14,20]. Tailoring guidelines to regional healthcare system capacities is essential to promote equity and sustainability [7,11], as well as to enhance CVD prevention and optimise resource allocation globally [6,12,13].

## CONCLUSIONS

Global statin guidelines must evolve to reflect both the structural diversity of healthcare systems and the clinical complexity of ageing. In older adults, clinical decisions should consider frailty, functional status, polypharmacy, and life expectancy, balancing benefits against competing risks and aligning with patient goals. In low-resource settings, statin use must be integrated into tiered, context-sensitive models that leverage task sharing, fixed-dose combinations, and simplified protocols. Updating global guidelines for statin use is clinically and ethically imperative to ensure equitable, feasible, age-appropriate, and person-centred CVD prevention.
